# Correction: No population bias to left-hemisphere language in 4-year-olds with language impairment

**DOI:** 10.7717/peerj.507/correction-1

**Published:** 2017-01-19

**Authors:** Dorothy V.M. Bishop, Georgina Holt, Andrew J.O. Whitehouse, Margriet Groen

**Affiliations:** 1 Department of Experimental Psychology, University of Oxford, UK; 2 Telethon Institute of Child Health Research, University of Western Australia, Perth, WA, Australia; 3 Radboud University, ED Nijmegen, Netherlands

The authors found an error in the program used that computed laterality indices from the functional transcranial Doppler ultrasound. This affects reported data and analysis which have been corrected

in this text and does not affect the methods and discussion.

