# Supplementary material for: No population bias to left-hemisphere language in 4-year-olds with language impairment
Source: PeerJ. 2014 Aug 7;2:e507. doi: 10.7717/peerj.507 (PMC4137668; doi:10.7717/peerj.507)

Supplementary figure for Bishop, D. V. M., Holt, G., Whitehouse, A. J. O., & Groen, M. No population bias to left-hemisphere language in 4-year-olds with language impairment. Error bars show 95% confidence interval for laterality index.


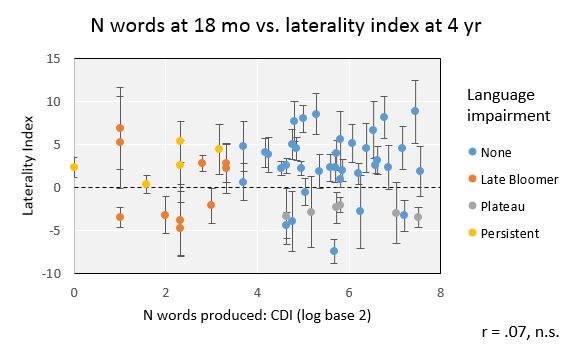

Supplement: Supplemental Information 1 [file peerj-02-507-s001.docx]
