# Supplementary material for: Correction: No population bias to left-hemisphere language in 4-year-olds with language impairment
Source: PeerJ. 2017 Jan 19;2:e507/correction-1. doi: 10.7717/peerj.507/correction-1 (PMC5255983; doi:10.7717/peerj.507/correction-1)
Supplement: Supplementary file 1 [file corrigendum-507.pdf]

This corrected version was necessary because an error was found in the program that computed laterality indices from the functional transcranial Doppler ultrasound. The laterality index should have been based on all administered trials. To estimate the reliability of this index, we also computed the same index using just the odd or even trials. A scripting error meant that the laterality index based on just the even trials had overwritten the index based on all trials. Thus the results originally reported were based on correct data, but used a laterality index based on only half the available trials.
The program and analysis have been corrected – these files are available on https://osf.io/yv4ra/.
In the corrected paper, the title, abstract, introduction, methods and discussion are all unchanged.

Changes are as follows:
Table 1 originally showed nonsignificant group differences on the LI; some mean values have changed from the original, but these are still nonsignificant.
Table 2 also showed a whole series of measures on which two groups did not differ significantly. This is still the case, but for this table, all the mean values are now changed from the original, because group membership was determined by LI and this changed for some cases.
Table 3 showed two ways of analysing LI data in relation to language impairment – one categorical and one quantitative. In the original, the categorical measure was nonsignificant and the quantitative was significant at the .05 level – this has now flipped, so the categorical is significant and the quantitative is nonsignificant. The main finding, that the language-impaired children show no evidence of bias to the left, whereas the typical children show substantial bias is reported in the text and is closely similar to the original result.
The text also reports some correlations that were computed to confirm that factors such as number of trials and mean length of utterance did not explain the group difference: these are only slightly different from the original version.
Figure 2, which shows individual data for LI in a beeswarm plot for four groups, is also slightly different, reflecting small changes to the LI for individuals. The same is true of the supplementary Figure, which shows the scatterplot relating LI to a measure of vocabulary in toddlers.

Textual changes in the Results section are as follows:

Original text:

There is no significant difference in the proportions with left-, bilateral and right-hemisphere language in these two groups, but on the quantitative laterality index (LI), the difference between the two groups was significant with effect size (Cohen's d) of 0.76. For those with no language difficulties at 4 years, there was a significant bias to left-sided language, t (45) = 3.83, p < .001, whereas for those with language difficulties at 4 years, the mean laterality index did not differ significantly from zero, t (10) = -0.29, p = .779.

Before accepting these results at face value, it is necessary to consider possible confounding factors that might lead to a spurious association. First, we asked whether children with current language difficulties might show weaker laterality because they completed fewer trials and so had less reliable data. This did not seem the case. Although the children with language difficulties completed fewer trials on average on the fTCD procedure, this could not explain their reduced asymmetry, as the number of trials did not correlate significantly with the LI, Pearson r = -.007, p = .958. Furthermore, the split-half reliability of the LI, calculated from the intraclass correlation between odd and even trials, was high and closely similar for the two groups. Also, the trend for weaker

right-handedness in the language-difficulties group on the handedness inventory could not account for the LI difference, since this variable did not correlate significantly with LI either, Spearman’s rho = .17, N = 57, p = .209. Overall, the majority of children in both groups were right-handed for writing: 39 of 46 (85%) of those with no language difficulties and 9 of 11 (90%) of those with language difficulties, a non-significant difference, c2 = 0.3, d.f. = 1, p = .859. (One case had missing data). Thus it does not seem possible to explain the high rate of atypical language laterality in the language-difficulties group either in terms of less reliable measurement, or because of adventitious inclusion of non-right-handers.

An important question is whether reduced left-hemisphere lateralization is seen because the group with language difficulties produced fewer words during the fTCD activation procedure. The difference between groups was significant for mean length of utterance, but neither this variable, nor the number of utterances, correlated significantly with the LI; Pearson r = .12, p = .373 for number of utterances, and r = -.01, p = .960 for mean length of utterance. Note too that we excluded trials where the child said nothing during the activation procedure.

Because of the small sample size, we had grouped together all children with language difficulties at 4 years, regardless of their status at 20 months. It is of interest, nevertheless, to explore the data further to see whether reduced lateralization is a particular characteristic of those with current language difficulties, or whether it is also associated with past history of language delay.

Figure 2 shows relevant data. This shows that atypical lateralization is also seen in late bloomers, i.e., those who had been identified as late-talkers at 20 months of age, but subsequently improved. Four of these ten children had right hemisphere language and one had bilateral language. An unexpected finding was obtained when we subdivided the 'language difficulties' 4-year-olds into those who did or did not have earlier language delay. For those who had not been late talkers, referred to here as cases of language ‘plateau’, all six had right hemisphere (N = 3) or bilateral (N = 3) language. In contrast, language-impaired 4-year-olds who had been late talkers had predominant left-lateralization of language, just like the typically-developing children who had never had language difficulties. However, the numbers are very small and it is possible that this was a chance effect. In general, the data are consistent with the view that in a group of children who have either early language delay and/or language impairment at 4 years there is no overall bias to left-sided language lateralization. Figure 2 also shows data on handedness, confirming the lack of close relationship between writing hand and LI on the story-description task.

Corrected text:

There was a significant difference in the proportions with left-, bilateral and right-hemisphere language in these two groups, though on the quantitative laterality index (LI), the difference between the two groups fell short of significance. For those with no language difficulties at 4 years, there was a significant bias to left-sided language, t (45) = 3.80, p < .001, whereas for those with language difficulties at 4 years, the mean laterality index did not differ significantly from zero, t (10) = -0.20, p = .850.

Before accepting these results at face value, it is necessary to consider possible confounding factors that might lead to a spurious association. First, we asked whether children with current language difficulties might show weaker laterality because they completed fewer trials and so had less reliable data. This did not seem the case. Although the children with language difficulties completed fewer trials on average on the fTCD procedure, this could not explain their reduced asymmetry, as the number of trials did not correlate significantly with the LI, Pearson r = .105, p = .438. Furthermore, the split-half reliability of the LI, calculated from the intraclass correlation between odd and even trials, was high and closely similar for the two groups. Also, the trend for weaker right-handedness in the language-difficulties group on the handedness inventory could not account for the LI difference, since this variable did not correlate significantly with LI either, Spearman's rho = .196, N = 57, p = .143. Overall, the majority of children in both groups were right-handed for writing: 39 of 46 (85%) of those with no language difficulties and 9 of 11 (90%) of those with language difficulties, a non-significant difference, χ^2 = 0.3, d.f. = 1, p = .859. (One case had missing data). Thus it does not seem possible to explain the high rate of atypical language laterality in the language-difficulties group either in terms of less reliable measurement, or because of adventitious inclusion of non-right-handers.

An important question is whether reduced left-hemisphere lateralization is seen because the group with language difficulties produced fewer words during the fTCD activation procedure. The difference between groups was significant for mean length of utterance, but neither this variable, nor the number of utterances, correlated significantly with the LI; Pearson r = 11, p = .399 for number of utterances, and r = -.03, p = .852 for mean length of utterance. Note too that we excluded trials where the child said nothing during the activation procedure.

Because of the small sample size, we had grouped together all children with language difficulties at 4 years, regardless of their status at 20 months. It is of interest, nevertheless, to explore the data further to see whether reduced lateralization is a particular characteristic of those with current language difficulties, or whether it is also associated with past history of language delay.

Figure 2 shows relevant data. This shows that atypical lateralization is also seen in late bloomers, i.e., those who had been identified as late-talkers at 20 months of age, but subsequently improved. Four of these ten children had right hemisphere language and three had bilateral language. An unexpected finding was obtained when we subdivided the 'language difficulties' 4-year-olds into those who did or did not have earlier language delay. For those who had not been late talkers, referred to here as cases of language 'plateau', all six had right hemisphere (N = 4) or bilateral (N = 2) language. Language-impaired 4-year-olds who had been late talkers had predominant left-lateralization of language (3 left and 2 bilateral). However, the numbers are very small and it is possible that this was a chance effect. Typically-developing children who had never had language difficulties included 26 left-lateralized, 6 bilateral and 4 right-lateralized cases). In general, the data are consistent with the view that in a group of children who have either early language delay and/or language impairment at 4 years there is no overall bias to left-sided language lateralization. Figure 2 also shows data on handedness,

confirming the lack of close relationship between writing hand and LI on the story-description task.
